# Prenatal Diagnosis by Trio Clinical Exome Sequencing: Single Center Experience

**DOI:** 10.3390/cimb46040201

**Published:** 2024-04-06

**Authors:** Katia Margiotti, Marco Fabiani, Antonella Cima, Francesco Libotte, Alvaro Mesoraca, Claudio Giorlandino

**Affiliations:** 1 Human Genetics Lab, Altamedica Main Centre, Viale Liegi 45, 00198 Rome, Italy; marco.fabiani@artemisia.it (M.F.); antonella.cima@artemisia.it (A.C.); francesco.libotte@artemisia.it (F.L.); alvaro.mesoraca@artemisia.it (A.M.); claudio.giorlandino@artemisia.it (C.G.); 2 Fetal-Maternal Medical Centre, Altamedica Viale Liegi 45, 00198 Rome, Italy

**Keywords:** fetal anomalies, clinical exome sequencing, chromosomal microarray analysis, prenatal diagnosis

## Abstract

Fetal anomalies, characterized by structural or functional abnormalities occurring during intrauterine life, pose a significant medical challenge, with a notable prevalence, affecting approximately 2–3% of live births and 20% of spontaneous miscarriages. This study aims to identify the genetic cause of ultrasound anomalies through clinical exome sequencing (CES) analysis. The focus is on utilizing CES analysis in a trio setting, involving the fetuses and both parents. To achieve this objective, prenatal trio clinical exome sequencing was conducted in 51 fetuseses exhibiting ultrasound anomalies with previously negative results from chromosomal microarray (CMA) analysis. The study revealed pathogenic variants in 24% of the analyzed cases (12 out of 51). It is worth noting that the findings include de novo variants in 50% of cases and the transmission of causative variants from asymptomatic parents in 50% of cases. Trio clinical exome sequencing stands out as a crucial tool in advancing prenatal diagnostics, surpassing the effectiveness of relying solely on chromosomal microarray analysis. This underscores its potential to become a routine diagnostic standard in prenatal care, particularly for cases involving ultrasound anomalies.

## 1. Introduction

Fetal anomalies are structural or functional abnormalities that occur during intrauterine life. According to the World Health Organization (WHO), every year about 300,000 newborn babies die worldwide within 28 days of birth due to congenital disorders (https://www.who.int/news-room/fact-sheets/detail/birth-defects (accessed on 2 April 2024)). Etiology refers in part to a genetic abnormality as a cause, i.e., chromosomal abnormalities, or single gene defects (for example, cystic fibrosis) [1]. Congenital heart malformations, cleft lip or palate, club foot, and other congenital illnesses with unclear causes are thought to be the result of complex genetic and environmental interactions. Consanguinity also increases the prevalence of rare genetic congenital disorders and nearly doubles the risk of neonatal and childhood death, intellectual disability, and other anomalies. Current guidelines recommend a cytogenetic karyotype followed by chromosomal microarray analysis (CMA) as a first-tier prenatal test in cases of ultrasound fetal anomalies [2]. The CMA identifies a causal diagnosis in around 6% of fetuseses with ultrasound anomalies and a normal karyotype [3]. Recently, new data have emerged that further supports the use of whole exome or clinical exome sequencing in prenatal diagnosis [4,5]. Clinical exome sequencing (CES) is a powerful diagnostic tool used to analyze the protein-coding regions. CES involves sequencing these critical regions to identify genetic variations that may be responsible for a patient’s symptoms or underlying condition [6]. These data demonstrate an increased rate of successful diagnosis in fetal samples obtained through amniocentesis and chorionic villus sampling (CVS) after negative CMA results [7,8,9,10]. In this study, we carried out in a trio exome analysis setting (proband and both parents) a clinical exome sequencing (CES) approach to identify the possible genetic cause after ultrasound anomaly detection. We compared the coding regions of all clinically relevant genes included in a commercial panel (Trusight One panel, Illumina; and more recently, the Trusight One Expanded panel, Illumina) between the parents and fetuses. After identifying possible causative pathogenic variations using variant filtering approaches, bioinformatic analysis was employed to choose the most relevant genetic variants according to their significance in association with the genotype and the disease phenotype. Reference databases, such as Human Gene Mutation Database (HGMD) professional (accessed on 1 January 2018), Online Mendelian Inheritance in Man (OMIM), Ensembl, ClinVar (NCBI), Varsome and American College of Medical Genetics (ACMG), were updated to the date of collecting samples. Trio analysis permitted us to investigate the origin of every fetal variation and assess its possible causal role using the concepts of familiar segregation. Recently, the implementation of clinical exome sequencing based on the use of trio analysis has shown a high diagnostic yield [8,11,12]. The aim of our study was to evaluate the feasibility of increasing the diagnostic yield by trio clinical exome in CMA negative samples.

## 2. Materials and Methods

### 2.1. Patients

The study includes the investigation of 51 pregnant women who were admitted at Altamedica Medical Centre (Rome, Italy) and underwent a fetal prenatal test between January 2018 and October 2023. The study was approved by the local ethical committee, Artemisia SPA (Approval Code: #008-2017-012, 3 December 2017), and all participants were provided with written informed consent. Pregnancies (11–28 weeks of gestation) were included following the detection of ultrasound anomalies and chromosomal microarray analysis negative results. Out of the 51 probands, 23 were male and 28 were female (Table 1). Fetal DNA was obtained through amniocentesis or Chorionic Villus Sampling (CVS).

### 2.2. CMA and Trio Clinical Exome Sequencing Analysis

The fetal and parental DNA was submitted for trio clinical exome diagnostics after excluding fetal aneuploidy, structural defects by classical karyotyping and microdeletion/microduplication syndromes by chromosomal microarray analysis. Genomic DNA was extracted from amnio or villus sampling and from the parental peripheral blood using the DNeasy Blood & TissueKit and QIAamp DNA Blood Mini Kit, according to the manufacturer’s instructions (Qiagen, Hilden, Germany). The CMA was performed using 44 K or 60 K platforms on DNA extracted from amniotic fluid or CVS to characterize the presence of the DNA deletions or duplications, following the manufacturer’s protocol (Agilent Technologies, Santa Clara, CA, USA). Trio clinical exome sequencing was conducted using the TruSight One Sequencing Panel until October 2020, and from November 2021 until the end by TruSight One expanded Sequencing Panel, according to the manufacturer’s instructions (Illumina, San Diego, CA, USA). The first panel covers 4813 disease-associated genes, and the second panel covers 6840 disease-associated genes. The targeted exonic regions underwent paired-end sequencing on an Illumina platform, using a NextSeq 550Dx sequencing system (Illumina, San Diego, CA, USA). The variants were considered pathogenic and likely pathogenic based on ClinVar, a freely accessible public archive (https://www.ncbi.nlm.nih.gov/clinvar/intro/) (accessed on 1 January 2018), ACMG recommendations, Varsome and Human Gene Mutation Database (HGMD), available via http://www.hgmd.org (accessed on 1 January 2018) [13], and the results were returned to the referring clinicians. Furthermore, by using bioinformatic tools such as eVai software (https://www.engenome.com, v3.1) (accessed on 2 April 2024), Geneyx software (https://analysis.geneyx.com/#/, v 5.16) (accessed on 2 April 2024) on the exome sequencing results, we were able to prioritize variants related to the ultrasound anomalies. Each fetal anomaly was labelled according to HPO terminology with HP identifiers [14,15].

## 3. Results and Discussion

Between January 2018 and October 2023, 51 pregnant women were admitted to Altamedica Medical Centre in Rome, Italy, for fetal prenatal testing subsequent to the detection of fetal abnormalities during ultrasound examinations (Table 1). None of the 51 couples were consanguineous. Fetal ultrasound showed at least one anomaly, including a wide spectrum of signs ranging from nuchal translucency measurement ≥ 3.0 mm in 18/51 pregnancies (35%), brain malformation in 13/51 pregnancies (25%), heart abnormalities in 9/51 pregnancies (18%), polyhydramnios in 6/51 pregnancies (11%), and skeletal anomaly in 5/51 pregnancies (10%) (Table 1). Once macroscopic chromosomal aneuploidies or rearrangement anomalies were excluded by classical cytogenetics karyotyping, CMA analysis was performed, using 44 K or 60 K platforms (Agilent Technologies) on DNA from liquid amniotic or CVS samples, to characterize the presence of microdeletions or microduplications. After negative CMA, trio clinical exome sequencing by next generation sequencing (NGS) was performed on the fetuses and their parents’ genomic DNA. In total, trio clinical exome identified a causative molecular diagnosis (pathogenic variants or likely pathogenic variants) in 12/51 fetuses (24%), leading to pregnancy termination in 5/12 (after molecular results) (Table 2).

Pathogenic variants involved in recessive disorders were identified in 5/12 fetuses (42%), which included a compound heterozygous pathogenic variant in *GALC* gene (c.379C>T; c.863G>A) and four homozygous inherited pathogenic variants from unaffected carrier parents in *ASPM*, *DHCR7*, *PEX1* and *CC2D2A* genes (Table 2). Pathogenic variants involved in autosomal dominant disorders were identified in 7/12 (58%) fetuses, which included six de novo heterozygous causative genetic variants in *EXT2*, *COL1A1*, *SIX3*, *HRAS* and *PTPN11* genes and one inherited pathogenic variant from healthy parents in *EXT1* gene (Table 2). Secondary anomalies were further added by the clinicians for the solved cases when a review of imaging was possible (Table 2).

**Figure 1:**
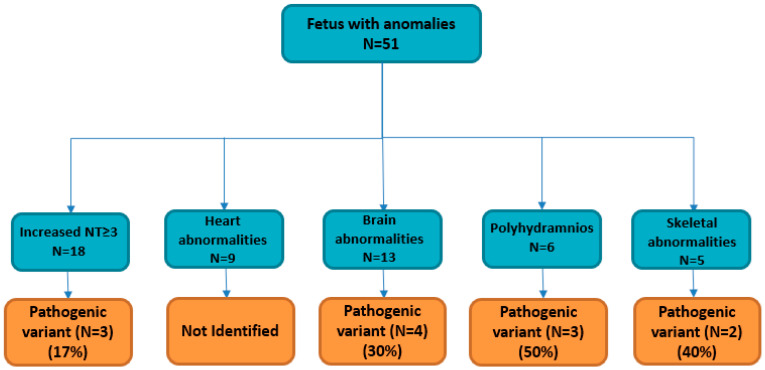
Fetuses with ultrasound anomalies included for clinical exome analysis, and solved cases for each fetal phenotype.

The diagnostic yield was the highest in the polyhydramnios subgroup patients (3/6), with positive CES results reaching 50% of the diagnostic yield (Table 1, Figure 1).

The presence of polyhydramnios may indicate the possibility of a neurological disorder, congenital malformation, or renal disorder that can disrupt the balance of amniotic fluid [16]. An underlying genetic condition has been observed in 3% to 13% of pregnancies with signs of polyhydramnios ultrasound anomalies [17,18]. The trio clinical exome sequencing on fetuses with a polyhydramnios fetal phenotype revealed, in three out of six cases, a likelily causative pathogenic variant in *PTPN11* and *HRAS* genes (Table 2). *HRAS* mutation (p.Gly13Cys) was previously identified in a patient with a clinical diagnosis of Costello syndrome (CS; MIM 218040) [19], while *PTPN11* pathogenic variants (c.174C>G, and c.923A>G) were previously identified in two distinct patients with a clinical diagnosis of Noonan syndrome (NS, MIM 163950) [20] (Table 2). Noonan and Costello belong to a group of spectrum disorders named RASopathies, which are phenotypically similar congenital anomaly disorders associated with pathogenic variants in genes of the Ras/MAPK signalling pathway, a cascade critical for the regulation of cell proliferation and differentiation [20,21]. Polyhydramnios is a very common sign in RASopathies, generally in combination with other evocative ultrasound findings, but it has even been reported as a unique fetal ultrasound anomaly [22,23]. 

In our cohort, the most frequent soft marker was an increased NT, and 18 pregnant women entered into care with a routine ultrasound examination revealing a higher NT (NT ≥ 3.0 mm) (Table 1, Figure 1). Increased NT thickening serves as a marker for several genetic abnormalities, such as aneuploidy, structural defects (cardiac anomalies, cleft lip/palate), microdeletion/microduplication syndromes, single-gene disorder syndromes (Noonan, Smith–Lemli–Opitz, Escobar, Pena-Shokeir) or adverse pregnancy outcomes (miscarriage, intrauterine death, perinatal death, birth defects) [24,25,26,27].

After excluding fetal aneuploidy, structural defects and microdeletion/microduplication syndromes, the 18 pregnant women with increased NT were enrolled in the trio clinical exome sequencing study (Figure 1). Three out of eighteen fetuses showed pathogenic variants in causative gene disease (*EXT1*, *EXT2*, and *DHCR7*). Two heterozygous pathogenic variants (p. Trp606Ter in *EXT1* gene, and p.Y143Ter in *EXT2* gene) are responsible for an autosomal dominant disease, Hereditary Multiple Exostoses (HME; MIM 133700, 133701) [28] (Table 2). It is a rare orphan disease with an unknown exact incidence due to asymptomatic individuals who remain undiagnosed [29]. *EXT1* (8q24.11-q24.13) and *EXT2* (11p12-p11) are two tumor suppressor genes that encode for glycosyltransferases involved in the synthesis of heparan sulphate proteoglycans; heterozygous single nucleotide variants, deletions, or duplications in these genes resulting in frameshifts or loss of *EXT1* and *EXT2* expression are identified in approximately 80% of patients with HME. In our cohort, in fetus 5, we detected a homozygous nonsense mutation c.453G>A (p.W151X) in the *DHCR7* gene (Table 2). Pathogenic variants identified in the *DHCR7* gene are responsible for Smith–Lemli–Opitz syndrome (SLOS OMIM #270400), an autosomal recessive metabolic disorder affecting the last step of cholesterol synthesis [30]. SLOS remains undiagnosed in many affected fetuses due to variations in fetal phenotypes and a lack of experience with fetal syndromology. SLOS prenatal ultrasound diagnosis showed internal malformations, including heart anomalies, renal anomalies ranging from renal hypoplasia to uni- or bilateral renal agenesis, and cerebral malformations. Interestingly, further detailed ultrasound information obtained from clinicians on the fetus 5 phenotype indicates a renal anomaly in addition to the increased NT [5] (Table 2). Brain malformations, such as holoprosencephaly, microcephaly, and ventriculomegaly, were reported by the clinicians during ultrasound assessment. Thirteen fetuses referred with brain malformations were selected for trio clinical exome after excluding fetal aneuploidy, structural defects and microdeletion/microduplication syndromes (Figure 1). Four out of thirteen fetuses showed pathogenic variants in the causative disease gene (*SIX3*, *ASPM*, *PEX1*, and *CC2D2A*). Two ventriculomegaly fetuses showed causative mutations in *PEX1* gene and *CC2D2A* gene (Table 2). Fetal ventriculomegaly is one of the most frequently diagnosed abnormalities of the central nervous system (CNS) on ultrasonography examination. Specifically, PEX1 is one of the genes associated with Zellweger spectrum disorders (ZSDs), a rare autosomal recessive disorder causing developmental delays, intellectual disability, hypotonia (weak muscle tone), feeding difficulties, vision and hearing impairment, and facial abnormalities [31]. *CC2D2A* mutations are a relatively common cause of Joubert syndrome, a severe disorder characterized by multiple congenital abnormalities, including kidney cysts, liver fibrosis, polydactyly (extra fingers or toes), and central nervous system malformations [32]. Holoprosencephaly and microcephaly are both congenital conditions that can affect brain development, but they involve distinct abnormalities. In these selected cases, the holoprosencephaly phenotype was associated with a pathogenic mutation in SIX3 gene, and the microcephaly phenotype was associated with a pathogenic mutation in *ASPM* gene (Table 2). Regarding *SIX3* gene, it has been shown that it plays a crucial role in the development of the forebrain and has been reported as a common cause of holoprosencephaly due to single-gene mutations [33]. Mutations in the ASPM gene have been extensively associated in the literature to a microcephaly phenotype [34]. Finally, skeletal abnormalities, such as a bilateral cleft lip/palate and short fetal femur length, were associated with previously reported pathogenic mutations in *GALC* gene and *COL1A1* gene (Table 2). Cleft lip and palate is one of the most frequent congenital anomalies, with an incidence of approximately 1 in 500–1 in 1000 live births [35]. In fetus 1, cleft and lip palate presented by the clinician as a primary ultrasound fetal phenotype, was associated with a major severe condition, Krabbe disease (OMIM 245200), due to heterozygote compound pathogenic mutations in GALC gene (p.Arg127Ter; p.Trp288Ter) that were inherited from the mother and father, respectively [36]. Krabbe disease is a rare (1 in 100.000 live births) and severe fatal autosomal recessive neurodegenerative disorder characterized by progressive neurologic deterioration resulting in death before the age of 2 years in most affected infants. Interestingly, only one previous study found this rare association between fetal cleft lip and palate and Krabbe disease [37]. 

The other skeletal abnormalities were associated with the well-known disease osteogenesis imperfecta type I, due to a pathogenic mutation in *COL1A1* gene (c.2684dupC). All genetics variants were previously reported as pathogenic or disease-causing mutations in ClinVar, a freely accessible public archive (https://www.ncbi.nlm.nih.gov/clinvar/intro/) (accessed on 1 January 2018), and/or Human Gene Mutation Database (HGMD^®^), available via http://www.hgmd.org (accessed on 1 January 2018). 

The diagnostic yield was the highest in the polyhydramnios subgroup, with positive clinical exome sequencing analysis in 3/6 (50%), and the lowest diagnostic yield was in the increased nuchal translucency subgroups (17%), demonstrating that in our cohort, the identification of a fetal ultrasound phenotype such as polyhydramnios was more evocative of an underlying genetic condition than increased nuchal translucency ≥ 3 mm (Figure 1).

With a diagnostic yield of 24%, this study underscores the effectiveness of trio clinical exome analysis in identifying causative genetic variants underlying a diverse spectrum of anomalies observed on fetal ultrasound examinations. The integration of NGS data from trio analysis into prenatal diagnostics not only provides valuable insights into the etiology of developmental abnormalities but also facilitates informed decision-making for parents and clinicians regarding pregnancy management and potential therapeutic interventions.

Furthermore, the identification of pathogenic variants associated with both recessive and dominant disorders highlights the genetic heterogeneity underlying fetal anomalies and underscores the importance of comprehensive genetic testing approaches in capturing the full spectrum of causative variants. The detection of de novo heterozygous variants, as well as inherited pathogenic variants from carrier parents, emphasizes the need for thorough genetic counseling and cascade testing within families to assess the risk of recurrence and provide appropriate support and guidance. Importantly, the differential diagnostic yield observed across various ultrasound phenotypes underscores the utility of specific ultrasound markers, such as polyhydramnios, in predicting underlying genetic conditions and guiding the selection of targeted genetic testing strategies. The higher diagnostic yield observed in the polyhydramnios subgroup highlights the clinical relevance of this ultrasound finding as a potential indicator of genetic abnormalities, prompting further investigation with trio clinical exome sequencing.

Moreover, the identification of VUS underscores the complexity of variant interpretation and the need for ongoing research and collaboration with clinicians to elucidate their clinical significance and pathogenicity. The inclusion of minimal ultrasound information in genetic reports emerges as a critical aspect of variant interpretation, facilitating the prioritization of variants and enhancing the diagnostic yield of genetic testing.

Overall, this study underscores the transformative impact of genomic medicine in prenatal diagnostics, offering unprecedented opportunities for early detection, accurate diagnosis, and personalized management of congenital malformations. With the development of genetic testing technologies and their accessibility for everyone, genomic data integration into prenatal care becomes a new way of improving the prognosis of those fetuses that are affected and of empowering families to make an informed decision on their pregnancies by using the genetic information.

In conclusion, in this study, by applying a trio analysis in cases with a normal karyotype and aCGH, we obtain a diagnostic yield of 24%, showing that trio clinical exome analysis constitutes a pivotal instrument in the comprehensive assessment of congenital malformations identified during prenatal screening.

## Figures and Tables

**Table 1:** Detected ultrasound anomaly in 51 fetuses with negative CMA results included in the study.

Primary Ultrasound Anomaly	Total Cases	Solved Cases (Diagnostic Yield)
Increased NT ≥ 3 mm *	18	3 (17%)
Heart abnormalities	9	ND
Brain abnormalities	13	4 (30%)
Polyhydramnios	6	3 (50%)
Skeletal abnormalities	5	2 (40%)
**Fetal sex**		
Male	23	
Famele	28	

* NT = nuchal translucency measurement ≥ 3 mm. ND = Not Detected.

**Table 2:** Summary of ultrasound abnormalities, fetal phenotypes, zygosity, inheritance patterns, and related genetic conditions detected in 12 out of 51 prenatal trios analyzed.

Fetuses	Gene	Transcript Change	Protein Change	Primary Ultrasound Signs	Secondary Ultrasound Signs	Fetal Phenotype (HP **)	Zigosity	Transmission	ClinVar/HGMD *	OMIM Gene	Disease	OMIM Disease	Inheritance
1	*GALC*	c.379C>T; c.863G>A	p.Arg127Ter; p.Trp288Ter	Bilateral cleft lip/palate	NA	HP:0002744	*Compound heterozygosity*	*Biparental*	Path.	606890	Krabbe disease	245200	AR
2	*SIX3*	c.385G>T	p.Glu129Ter	Holoprosencephaly	NA	HP:0001360	*Heterozygosity*	De Novo	Path.	603714	Holoprosencephaly 2	157170	AD
3	*EXT2*	c.429C>G	p.Y143Ter	Increased NT = 3,0	NA	HP:0010880	*Heterozygosity*	De Novo	Path.	608210	Exostoses, multiple, type 2	133701	AD
4	*EXT1*	c.1818G>A	p.Trp606Ter	Increased NT = 3,0	NA	HP:0010880	*Heterozygosity*	*Paternal*	Path.	608177	Exostoses, multiple, type 1	133700	AD
5	*DHCR7*	c.453G>A	p.W151X	Increased NT = 3,5	Renal anomalies	HP:0010880; HP:0000077	*Homozygous*	*Biparental*	Path.	602858	Smith–Lemli–Opitz syndrome	270400	AR
6	*ASPM*	c.3055C>T	p.Arg1019Ter	Microcephaly	NA	HP:0000252	*Homozygous*	*Biparental*	Path.	605481	Microcephaly 5, primary, autosomal recessive	608716	AR
7	*HRAS*	c.37G>T	p.Gly13Cys	Polyhydramnios	Pleural effusion	HP:0001561; HP:0002202	*Heterozygosity*	De Novo	Path.	190020	Costello syndrome	218040	AD
8	*PTPN11*	c.174C>G	p.Asn58Lys	Polyhydramnios	NA	HP:0001562	*Heterozygosity*	De Novo	Path.	176876	Noonan syndrome 1	163950	AD
9	*PTPN11*	c.923A>G	p.Asn308Ser	Polyhydramnios	Renal anomalies	HP:0001563; HP:0000077	*Heterozygosity*	De Novo	Path.	176876	Noonan syndrome 1	163950	AD
10	*COL1A1*	c.2684dupC	p.Gly896fs	Short fetal femur length	NA	HP:0011428	*Heterozygosity*	De Novo	Path.	120150	Osteogenesis imperfecta, type I	166200	AD
11	*PEX1*	c.2528G>A	p.Gly843Asp	Ventriculomegaly	NA	HP:0002119	*Homozygous*	*Biparental*	Path.	602136	Heimler syndrome 1	234580	AR
12	*CC2D2A*	c.3850C>T	p.Arg1284Cys	Ventriculomegaly	NA	HP:0002119	*Homozygous*	*Biparental*	Path.	612013	Meckel syndrome 6	612284	AR

* Classified as disease-causing mutation (DM) on HGMD or as pathogenic on ClinVar; NT = Nuchal Translucency; NA = Not Available; AR = Autosomal Recessive; AD = Autosomal Dominant; ** Human Phenotype = HP.

## Data Availability

Data is unavailable due to privacy.
